# A cartridge based Point-of-Care device for complete blood count

**DOI:** 10.1038/s41598-019-54006-3

**Published:** 2019-12-09

**Authors:** Usama Abbasi, Prasanta Chowdhury, Sasikala Subramaniam, Prakhar Jain, Nitin Muthe, Faisal Sheikh, Subham Banerjee, V. Kumaran

**Affiliations:** 1 MicroX Labs, Society of Innovation and Development, Indian Institute of Science, Bangalore, 560012 India; 2 Department of Chemical Engineering, Indian Institute of Science, Bangalore, 560 012 India; 3 CSIR-National Aerospace Laboratory, HAL Airport Road, Bangalore, 560017 India

**Keywords:** Engineering, Biomedical engineering, Chemical engineering, Lab-on-a-chip

## Abstract

We demonstrate a proprietary lab-on-chip/*μ* TAS technology platform for a regulatory grade portable instrument for complete blood count (CBC) hematology tests including 3 part differential WBCs, RBCs, platelet and hemoglobin for rapid diagnostics at the point of care in resource-poor settings. Presently, diagnostics based on blood tests are confined to centralized laboratory settings, dependent on large footprint and expensive cytometers or on a microscope, requiring trained laboratory technicians. Consequently, such facilities are not present in rural and semi-urban settings, where there are opportunities and challenges in delivering efficient healthcare infrastructure at an affordable cost in resource-challenged environments. Our proposed design leverages advances in microfluidics and lab-on-chip fabrication techniques to miniaturize the conventional cytometer and bring down the cost significantly. The device can be operated autonomously, without skilled manpower, by primary healthcare professionals in the field and by patients (like glucose self-test devices). The instrument consists of a single-use chip, the size of a credit card, pre-loaded with reagents, in which the sample is loaded, and which is fluidically insulated from the environment. The controller, the size of a toaster, performs the necessary fluid handling and the impedance measurements to deliver the results in minutes.

## Introduction

Microfluidic devices hold forth the tantalising promise of integrating all the sample preparation and measurement functions of a diagnostic laboratory on to a small chip, thereby making laboratory diagnostic tests available at low cost in doctors’ clinics, at home and especially in resource challenged environments^1^. This promise has partially been realised in applications such as blood glucose monitors and pregnancy test kits, that do not involve complicated sample processing steps and where immunoassays can be used to test the presence or concentration of target molecules. In contrast, tests that require significant sample processing steps and enumeration of, for example, cells tagged with markers, have proved difficult to miniaturise and commercialise. The archetypal example of the latter is the Complete Blood Count (CBC) test, where it is necessary to count the numbers of the different types of blood cells in a blood sample. Attempts at creating a revolutionary low cost and miniature blood testing devices have proved to be illusory; the most prominent example is the spectacular downfall of a much-celebrated company, Theranos^2^. While companies working in the point-of-care medical devices have raised considerable amounts of capital in the past decade (at one point in time, Theranos was valued at 9 billion dollars apparently without even a working prototype), there has not been any commercial product that can carry out a test such as the CBC.

There appear many reasons for the failure of companies such as Theranos, including misrepresentation of results, inadequacy of oversight and the inability of investors to assess basic technological feasibility. However, there is the more fundamental question of how many tests can be conducted in a drop of blood^2^. Fundamental challenges in developing a miniature device for diagnostic tests such as blood cell counts include technical feasibility, difficulties in device integration, manufacturability/scale-up and cost^3^. Most of the research is focused on areas such as the study of flows in microfluidic devices, improving sensitivity of immunoassays, tagging cells and molecules with markers and signal processing for molecule/cell enumeration. While these are valuable advancements, the failure to develop microfludic point-of-care devices in spite of established demand and investment potential raises the issue of whether such devices are technologically feasible at all. The lack of peer-reviewed research from health-care startups has been cited as one of the reasons for the imperfect evaluation of purportedly disruptive technologies^4^. In order to engender confidence in the applicability of microfluidics for social benefit, it is important to demonstrate that a portable low-power low-footprint chip-based device, incorporating a range of technologies, can be used to autonomously carry out a sophisticated multi-parameter diagnostic tests. Here, we demonstrate a low-cost portable microfluidic chip based device for the widely used 14 parameter complete blood count (CBC).

The device consists of a disposable fluidic chip, the size of a credit card, pre-loaded with reagents. The blood sample from a fingerprick is loaded on to the chip. The chip is inserted into a controller the size of a toaster, which autonomously performs the sample preparation and cell enumeration and provides a read-out. Novel strategies are formulated for overcoming the two significant technological challenges related to the unit operations in sample preparation and the high-speed impedance-based cell counting. The cartridge is designed for ease of manufacturing by laser engraving and roll-to-roll processing, and we demonstrate the end-to-end integration of the different processes and technologies in a single device.

**Figure 1:**
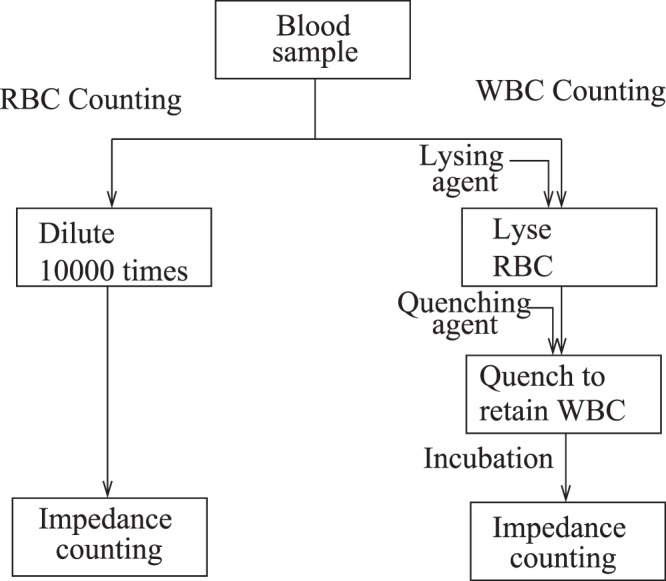
The sample preparation for the RBC and WBC counts.

One microliter of blood of a normal patient contains about 4.5–6 × 10^6^ red blood cells (RBCs/erythrocytes) which are disk-like with diameter about 8 *μ*m and thickness 2 *μ*m, about 4,000–10,000 white blood cells (WBCs/leukocytes) of size 10–20 *μ*m, and about 1.5–4.5 × 10^5^ platelets of size 2–3 *μ*m. In impedance-based cell counting, each cell passes in between electrodes embedded on opposite walls of a microchannel. The change in impedance due to the presence of the cell is used to count the number and type of cells that pass through the channel. For proper counting, it is essential to ensure the passage of only one cell at a time through the channel. This requires a 1:10^4^ dilution of the blood sample with Phosphate Saline Buffer (PBS 1×) to count the RBCs and the platelets. Since the RBCs and platelets have very different sizes, the difference in the impedance signals can be used to distinguish between them. However, the WBCs and the RBCs are comparable in size, and the WBCs are much smaller in number than RBCs. In order to count the WBCs, it is necessary to lyse the RBCs while preserving the WBCs. Since the WBCs are more resistant to lysing than the RBCs, a two-step process is used, where the RBCs are first lysed with a lysing agent, and a quenching agent is added in order to prevent lysing of the WBCs. After an incubation period for the degradation of the lysed blood cells, the sample is passed through an impedance sensor for enumeration. The sample preparation steps are summarised in Fig. 1.

Though the sample processing steps are easily carried out in a diagnostic laboratory, a similar process in a lab-on-a-chip device faces significant technological barriers^3^. Flows in microfluidic devices are usually laminar due to the small dimension and the low velocity; this is in contrast to sample preparation in a diagnostic laboratory which takes place due to turbulent mixing. The laminar flow in a microfluidic devices consists of parallel streamlines, and cross-stream mixing is due to molecular diffusion^5^. The following simple calculation demonstrates why the process of molecular diffusion is too slow for sample/reagent mixing in point-of-care applications. The molecular diffusion coefficient for small molecules such as nitrogen and oxygen in water is about D = 10^−9^ m^2^/s, while that for large and polymeric molecules could be as small as D ∼ 10^−13^ m^2^/s. Across a microchannel width of W ∼ 1  m, the diffusion time is (W^2^/D) ∼10^3^–10^6^ s. Therefore, diffusion across even a small distance in a microfluidic channel could take many tens of minutes; this is in contrast to turbulent mixing in a test tube in a diagnostic laboratory which is completed in seconds. Due to this, the path length of the microchannels in a microfluidic device are often many tens of centimeters long, even though the device itself is of a few centimeters in size^6,7^. A large pressure is required for pumping a fluid through a microchannel of small cross-section over a distance of tens of centimeters; this results in energy cost and the necessity for fabricating materials that can withstand the pressure. It is also a technological challenge to generate such large pressures repeatably in a low-footprint autonomous device, though such large pressures are easily generated using bulky syringe pumps in a laboratory setting.

Mixing strategies in microfluidic devices can be broadly separated into active strategies where external forcing is used to create time-dependent flows, and passive strategies, where tortuous flow paths, mix-and-recombine strategies or secondary flows are used to mix fluids. Secondary flows are generated by, for example, grooves etched into the walls a microchannel^8^, or circulation within droplets^9^. Other passive strategies include hydrodynamic focusing^10^, where the fluid stream is stretched in the stream-wise direction by a sheath flow, or split-and-recombine methods where the inlet stream is split and recombined multiple times in bifurcating and converging channels^11,12^. In active strategies, the fluid is mixed by microstirrers^13^, pressure waves^14^, acoustic pulses^15^ or electric fields^16,17^. Both of these strategies involve additional complexity and energy cost, either due to the enhanced pressure drop in flows with tortuous or fluctuating streamlines, or due to the mechanical/electrical/acoustic energy required for active mixing. Here, we use a novel, tunable, scalable and low energy cost strategy for ultra-fast mixing in a microchannel, by making one of the channel walls soft and elastic^18–21^.

The second technological challenge is the single cell impedance spectroscopy based on the contrast in the dielectric properties of water and of individual cells^22^. In conventional CBC, cell counting is carried out manually under a microscope after plating the cells. Image processing has also been used to develop automated optical counting, but examining a sufficiently large statistical sample is a cumbersome and time-consuming task. Flow impedance measurement is preferred over optics for CBC, since counting can be carried out at kHz frequency to count a statistically large sample in a few minutes.

In impedance counting, thin film electrodes deposited on the walls of a microchannel are used to record the change in impedance when a cell passes through the microchannel. The frequency of impedance signals due to the passage of cells provides the cell count, while the magnitude of the impedance can be used to sort the cell type. The change in impedance scales as the ratio of the cell volume and the volume of the microchannel^23^. Therefore, a robust impedance signal can be distinguished over the noise only if the volume of the microchannel is not much larger than the cell volume^24,25^. In the CBC, platelets are the smallest cells with size about 2–3 *μ*m, and it is desirable to have a measuring volume of that dimension for best resolution. However, channels of micron scale are liable to get blocked due to debris in real applications where thousands of samples pass through the sensor, though they may be useful for one-off measurements in the laboratory. To avoid channel blockage over extended time periods, the minimum channel dimension has to be at least 30 *μ*m. The localisation of the cell to micron dimensions within a much larger channel has been proposed using a sheath flow^26^, where the fluid containing the cell forms a thin film between two adjacent sheath fluid. However, this has the disadvantage of requiring complicated machining and a much larger volume of sheath fluid.

Repeatable sensing and identification of 2–3 *μ*m particles in a 30–40 *μ*m channel is a technical challenge; though it is desirable to have a high voltage to increase the signal-to-noise ratio, the maximum applied voltage is limited due to water electrolysis, breakdown and electrical safety. From the electrical safety perspective, the maximum voltage in a stand-alone device containing flowing liquids should be limited to about 15 V. The DC voltage for water electrolysis is 1.23 V, and AC voltages up to a few 100 kHz could also electrolyse water at voltages below 15 V to form nanobubbles^27^. Therefore, there is an upper bound on the voltage of about 15 V and a lower bound on the frequency much higher than 100 kHz.

Two-dimensional measurements at two different frequencies are necessary to distinguish different types of cells^28,29^. For this, it is necessary to generate two high frequency signals at a relatively low voltage, and then measure the impedance at the same frequencies. High frequency signal generation and frequency-locked measurements are usually carried out in high-cost Impedance analysers. Though these are common in research laboratories, they are too expensive and difficult to integrate in a low-footprint low-cost device. Here, we have developed a low-cost FPGA board using a 7 layer PCB stack for signal generation and frequency-locked measurements at 500 kHz and 2 MHz.

Semi-automatic sample preparation for CBC has been demonstrated in the past by van Berkel *et al*.^7^, but the system utilized bulky syringe pumps without any microfluidic valves, and a laboratory Impedance analyser. Carefully designed fluidic circuit performed the metering and dilution of the samples and the reagent. The reagent and the sample were loaded off-chip but the sample processing was done inside the cartridge. A technique that can be used for CBC at PoC with a single disposable cartridge with good accuracy that eliminates any human intervention has not been reported so far.

The major capabilities demonstrated here are as follows.

- Automatic sample preparation inside a single disposable cartridge the size of a credit card pre-loaded with reagent. The metering of the sample and the reagent is performed inside the cartridge in microchannels cut out in PSA (Pressure Sensitive Adhesive) by CO_2_ laser, which is ideally suited for low-cost roll-to-roll manufacturing.
- A microfluidic sensor with three pairs of top and bottom electrode to enumerate different type of cells without the necessity of sheath flow.
- The ability to distinguish between RBC, three different types of WBC and platelets using two-dimensional impedance measurement. A customised FPGA board based on a 7 layer PCB stack has been developed specifically for the signal generation and frequency-locked detection. The blood counts are validated against the industry-standard SYSMEX cytometer. We also demonstrate the ability to distinguish between polymer beads of 3 *μ*m, 4 *μ*m, and 5 *μ*m without the need for a sheath flow, and without the need correction algorithm for cell position in the cross-stream direction.
- Seamless end-to-end integration, including automatic sample processing, flow through the sensor, signal processing and enumeration for an automated device to carry out 14 parameter CBC from one drop of blood. All components have been integrated into an autonomous unit with footprint 15 cm × 11.5 cm with height 10 cm.

## Cartridge

**Figure 2:**
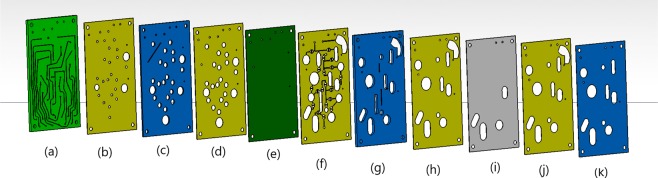
Burst view of the cartridge, from left to right, (**a**) airlines made on 2 mm thick acrylic sheet (**b**) PSA tape with access holes (**c**) 1 mm thin PMMA sheet (**d**) PSA tape (**e**) TPU membrane (**f**) PSA tape with fluidic channels (**g**) 5 mm thick PMMA sheet for reservoirs (**h**) PSA tape (**i**) PTFE membrane (**j**) PSA tape (**k**) 1 mm PMMA sheet.

The disposable cartridge consists of eleven layers stacked one on top of the other, as shown in the burst view in Fig. 2. The materials used are 1 mm, 2 mm and 5 mm Poly Methyl Methacrylate (PMMA) sheets from McMaster USA, Pressure Sensitive Adhesive (PSA) tapes of thickness 100 *μ*m, 3M-98010 PSA, a layer of Thermoplastic Polyurethane (TPU) membrane 3 M 9834 TPU of thickness 50 *μ*m, and a layer of hydrophobic Poly Tetra Fluoro Ethylene (PTFE) membrane of thickness 0.5 mm with pore thickness 1 *μ*m. The bottom five layers, (a–e), are the pneumatic manifold which contain the air lines and the pneumatic actuation elements for the valves and pumps. The air lines for controlling the valves and pumps are cut in the PMMA layer (a) of thickness 2 mm. The flexible TPU membrane layer (e) is used for opening/closing the valves, and serves as the diaphragm for the membrane pumps. The PTFE layer (i) is permeable to air but is impermeable to liquids, and so it is used for evacuating any air that may be trapped in the fluidic circuits. The fluidic channels and the fluid pathways in the valves and pumps are cut in the PSA layer (f) atop the TPU membrane. The width and height of the fluidic channels are 300 *μ*m and 100 *μ*m respectively. The reservoirs for the pre-loaded reagents, the buffer and the waste are cut in the PMMA sheet (g) of thickness 5 mm. The PMMA sheet (k) is used to cover the cartridge from above. The PSA layers (b), (d), (h) and (j) are used for bonding the adjacent sheets of different materials.

For fabricating the cartridge, 2D CAD designs of each layer were prepared. A CO_2_ Laser cutting machine with wavelength 10.6 *μ*m and maximum power 25 W made by Universal Laser Systems was utilized to precision cut all the eleven different layers needed for fabrication of a disposable cartridge. All the layers are laser cut by focusing a beam spot of size 25 micron on the substrate. After laser cutting, the liners are removed as necessary, and the layers are stacked using four alignment pins at the four corners. Then, the acrylic sheet and adhesive transfer tapes were passed through a laminator at 95 °C temperature for bonding and to remove any air trapped between them. After stacking all the layers, the cartridge was kept under 5 kg load for one hour to achieve better bonding. The stacked layers were visually inspected to ensure there are no air bubbles trapped inside. A quality check was performed on the pneumatic manifold by interfacing the access holes in the bottom layer with a pressure pump. A maximum 70 kPa pressure (in contrast to the 50 kPa pressure for operating the valves/pumps) was applied on the access holes for 5 minutes to check for the delamination of the TPU layer (layer (e)) with the fluidic channel (layer (f)). Visual inspection did not reveal any delamination of the TPU membrane with the fluidic channel or the PSA tape below.

**Figure 3:**
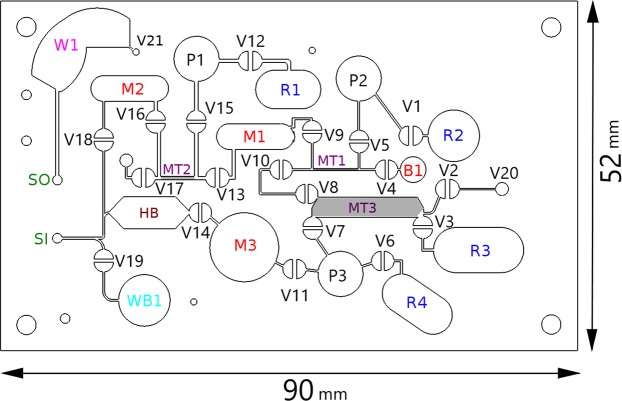
The cartridge layout consists of three membrane pumps P1-P3, twenty one valves V1-V21, four reservoirs for pre-loaded samples R1-R4, three mixing chambers M1-M3, three metering zones MT1-MT3, one reservoir for the blood sample B1, one waste buffer reservoir WB1, one waste reservoir W1, and one chamber HB for measuring hemoglobin content. SI and SO are the inlet and outlet ports for the flow to the sensor.

The layout of the microfluidic chip, shown in Fig. 3, consists of three membrane pumps, twenty one valves, four reservoirs for the pre-loaded reagents, three mixing chambers, three metering zones and reservoirs for the buffer, wash buffer and waste from the sensor. The reservoirs R1 and R2 are each filled with 300 *μ*l of Phosphate Buffer Saline (PBS) for dilution of the blood for processing the samples for counting RBCs, the reservoirs R3 and R4 are filled with the lysis reagent and quench reagent respectively for processing the sample for WBC counting. The composition of the lysis solution is 0.12% (v/v) formic acid, 0.05% (w/v) saponin, and the composition of the quench solution is 0.6% (w/v) sodium carbonate and 3% (w/v) sodium chloride. The inlet and outlet for the sensor are labeled SI and SO in Fig. 3.

The diameter of the on-chip pump P1 and P2 for RBC enumeration is 7 mm, and volume dispensed is about 2 ml for a cycle of 100 strokes. The diameter of the pump P3 for WBC enumeration is 5 mm, and the volume dispensed is 0.9 ml for a cycle of 100 strokes.The valves and on-chip membrane pump are opened and closed for 100 ms during suction and pumping phase. The stroke time of 100 ms was selected for all the pumps, and the variation in the volume dispensed by both pump is less than 3%.

Rapid mixing is required during the lysing of RBCs and the quenching during sample preparation for WBC enumeration. For this purpose, stirring is performed with magnetic flea of length 8 mm and diameter 1.5 mm in mixing chamber M3. The rotation of the flea was controlled by the DC motor having two strong magnets of opposite polarity stuck together on rotating disk, which was mounted on the shaft of the DC motor. The speed of the motor kept was 5000 RPM during the lysis of the erythrocyte to create a strong stirring effect in the third mixing chamber for removing the debris in the form of lysed erythrocytes.

The operation and control of the pumps and valves is explained in appendix A. The PMMA sheet (layer (a) in Fig. 2) contains 12 through holes at the location A1-A12 in Fig. 11 in the appendix A. The holes A1-A12 are connected to three-way solenoid valves (XValve 912–000001–003) manufactured by Parker which are used for switching pressure between +50 kPa and −50 kPa. The cartridge is kept on a platform with 12 ‘O’ rings which are press-fitted into slots on the base, using pressure applied from a clamp (Destaco, 305U). The details of the interfacing are given in appendix A. All the solenoid valves are connected to mechanical relays manufactured by HUIGANG (HRS2H-S-DC5V) which are switched on and off using a micro-controller. Two KNF (NMP830KNDC) pumps generate positive and negative pressure. The positive and negative pressures are regulated using two pressure regulator from SMC Pneumatics.

### Metering of blood sample

The institutional committee for approving experiments on human subjects at the National Aeronautical Laboratories (NAL), Bangalore, is the NAL ethics committee. This committee has approved the experiments, and all relevant guidelines and regulations were followed. Informed consent was obtained from all participants. 3 ml of blood was collected from healthy donors into VACUTAINER (K2 EDTA 5.4 mg) tube. Following the blood collection, the EDTA tubes were kept on a roller for continuous mixing of the blood and the EDTA salt. All the sample preparation was done within four hours after taking the venous blood from the donor.

The volume of the metering zones has to be calibrated carefully. If the volume is based on the design dimensions in the CAD drawing, the volume is overestimated. Due to incomplete laser cutting at the edges where the laser intensity decreases, the thickness of the metering zones in Fig. 3 is smaller than the design specification. Therefore, there is undercounting of number of cells per *μ*L if the volume is based on the CAD design. The volume of the metering zones was determined in two ways. One is by passing a suspension of spherical black polystyrene beads of diameter 6 *μ*m from Polysciences Inc., with 2.5% solids concentration. The suspension is passed through the fluidic circuit using procedures described in sections 2.2 and 2.3, and the number of particles in the output fluid streams are counted using a BD FACSVerse Flow Cytometer. The volume of the metering zone is determined from the particle count. The second method was to compare the total cell count (RBC + platelet for the RBC enumeration, and lymphocyte + monocyte + neutrophil count for the WBC enumeration) across all 6 patients from the present device and the SYSMEX counter, in order to calibrate the volume of the metering zone. Both of these methods gave results for the metering volume within 2%, and the volume determined by the latter procedure is used in our analysis.

In the metering operation, the blood sample is placed in the blood reservoir B1 in Fig. 3. The valves V2, V8, V10 and V4 are opened and all other valves are closed. A negative pressure is applied on valve V20, and blood is sucked in to the channels connecting the valves V4-V10-V8-V2. The 15 *μ*L of blood sample trapped in the metering zone MT3 between V8 and V2 is used for WBC counting. The 3 *μ*L of blood trapped in the metering zone MT1, which is the channel between the connections to valves V5 and V9, is used for RBC counting. Thereafter all the valves was closed. The filling of the blood sample inside the microchannel was significantly faster than the device with surface modification for passive capillary flow^30,31^. In passive capillary flow, the flow rate decreases with time, and fabrication is more complicated for a PoC device where the surface coating is required in the assembly line.

### Sample preparation for RBC & platelet enumeration

The RBC sample processing sequence is as follows. First the valves V1, V5 and V9 are opened and the pump P2 is operated to draw the 300 *μ*L of Phosphate Buffer Saline (PBS 1×) from the reservoir R2, along with the blood trapped in the metering zone MT1, into the mixing chamber M1. Thereafter, valves V13 and V17 are opened, and the pump P1 was operated to pump the diluted blood into the channel between the metering zone MT2. Valves V13 and V17 are closed, valves V12, V15 and V16 are opened, and pump P1 is used to pump 300 *μ*L of PBS from reservoir R1, along with the diluted sample trapped between V13 and V17, into the mixing chamber M2. The two sequential mixing steps result in 300 *μ*L of the blood sample of dilution 10^4^. In the subsequent counting step, valve V18 is opened and suction is applied on valve V21 to push the diluted blood through the sensor for RBC and platelet enumeration.

### Sample preparation for WBC enumeration

The lysis of RBCs for WBC enumeration is carried out using the third pump P3. The valves V3, V7 and V11 are opened. The pump P3 is operated to pump the blood in the metering zone MT3 between V3 and V7 into the mixing chamber M3, followed by the lysis solution in reservoir R3. The time taken for emptying the lysing solution into the mixing chamber is 10 s. After this, valves V3 and V7 are closed, valve V6 is opened and the quenching solution from reservoir R4 is pumped into the mixing chamber M3. During the lysis, the magnetic flea was rotated at more than 5000 RPM for rapid stirring. After an incubation time of 3 minutes, the valve V11 is closed. For enumeration, valve V14 is opened and suction is applied on valve V21 to pump the sample into the sensor through sensor inlet SI. In between, there is chamber HB with transparent walls which can be used for carrying out a hemoglobin concentration test using absorbance. The incubation time is necessary for the degrading the products of RBC lysis to a size below that detectable by the impedance sensor; if the incubation time is insufficient, the debris could be of sufficiently large size that they generate noise which could interfere with the impedance signal^32^. For our protocol, it is found that the debris does not interfere in the impedance signal if the incubation time is at least 3 minutes. The leukocytes remain intact for a period of 20 minutes after quenching, and so all the measurements are completed within this period. There is an additional effect of the lysis solution which is the chemical modification of the leukocyte membrane; the latter makes it possible to distinguish between the sub-populations of leukocytes using impedance measurements^33^. In our experiments, it is found that the magnetic stirring enhances the contrast in the impedance and opacity between the different sub-populations, and improves the quality of the enumeration.

## Sensor

**Figure 4:**
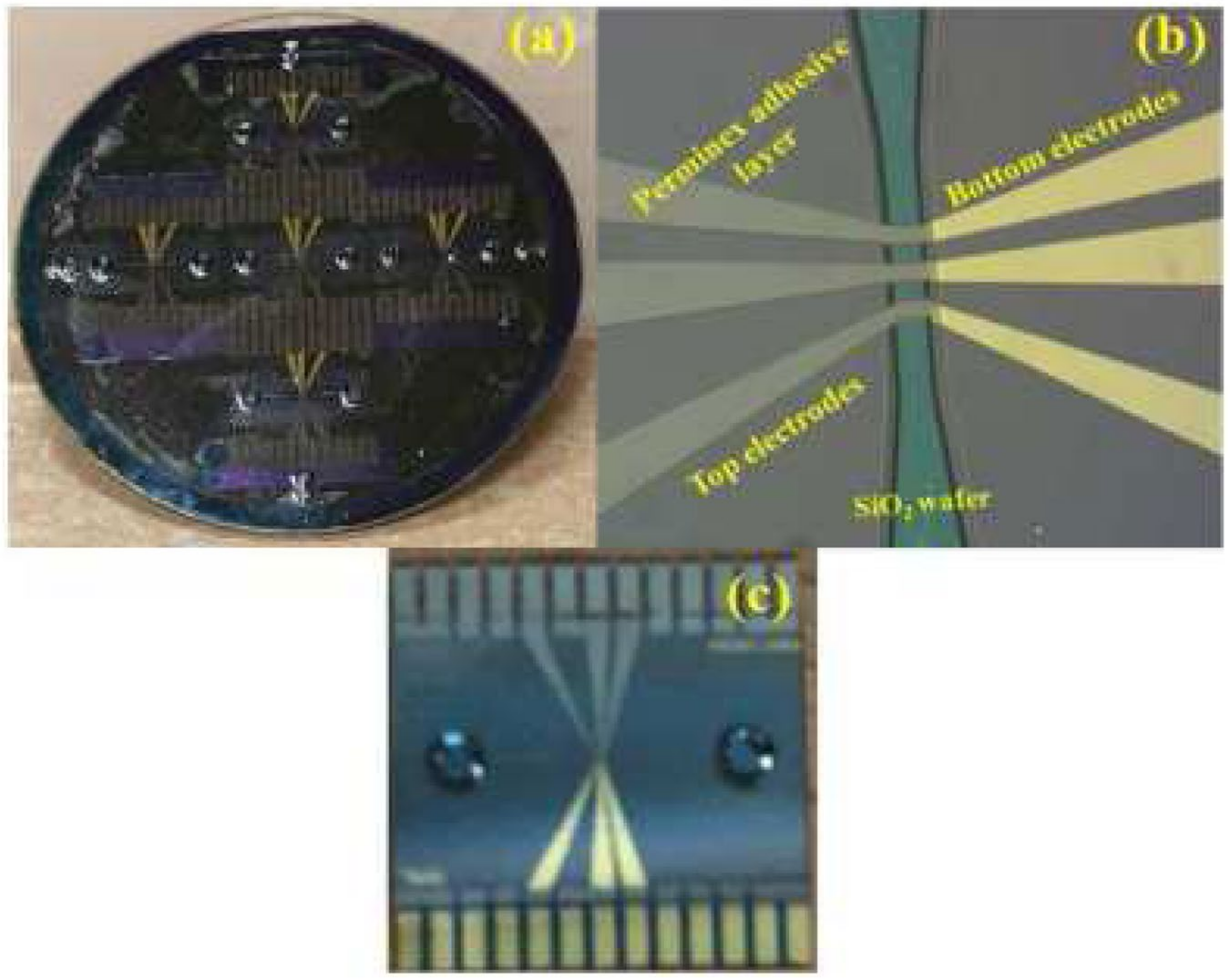
Images of the impedance sensor showing (**a**) bonded wafer, (**b**) fluidic channel with top and bottom electrodes and (**c**) diced impedance chip.

The microfluidic impedance sensor of dimension 13 mm × 13 m was fabricated using photolithography techniques, and a cost effective approach was made throughout the fabrication process. The fabrication process was divided into three components, (i) fluidic inlet/outlet ports, (ii) microelectrode fabrication, (iv) microfluidic channel fabrication followed by aligning and bonding of two wafers. In the microelectrode, the fluid flows between three pairs of electrodes deposited on an Si O_2_ coated Si wafer on one side and a glass wafer on the other side. A glass surface was chosen on one side, so that the fluid inlet and outlet could be etched into the surface. The microelectrodes comprise films of Ta(20 nm)/Pt(150 nm)/Au(50 nm) deposited on the two surfaces, and patterned on both wafers using a lift-off processes. The electrode width in the sensing region is 35 *μ*m and they are separated by a distance of 30 *μ*m along the surface. Through holes were made on the glass wafers using wet etching techniques. Photoimageable adhesive (Perminex, Microchem, USA) was coated on Si wafer and processed photolithographically to have the channel of width 45 *μ*m and height 30 *μ*m in the sensing region. Due to the adhesive nature of Perminex, both Si and glass wafers were softly bonded after alignment with an accuracy of ±1 *μ*m. The soft-bonded wafer transferred to a pressurized chamber and heated at around 120 °C for an hour. The thermally bonded wafer was then diced to single impedance chips, as shown in Fig. 4. O-rings are mounted on the inlet and outlet holes of the impedance sensor. When the cartridge is mounted in the control unit, the O-rings on the impedance sensor are pressed against the holes SI and SO on the microfluidic cartridge shown in Fig. 3. Details of the sensor fabrication are given in appendix B.

**Figure 5:**
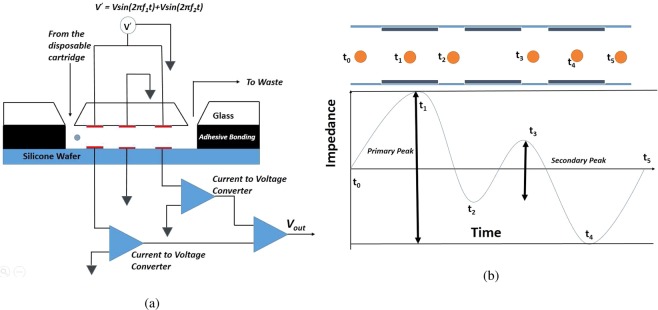
Schematic of the electrical circuit used for determining the change in impedance (**a**) and the change in the voltage signal as the cell passes in between the electrodes (**b**).

Figure 5(a) shows the schematic of the electrical circuit for measuring the change in the impedance. The cell passes sequentially between three pairs of electrodes, of which the central pair is grounded. A alternating voltage of 15 V peak-to-peak which is a combination of two different frequencies is applied to the top electrode in the upstream and downstream pair of electrodes, while the bottom electrodes are connected to the non-inverting input of two trans-impedance operational amplifiers (THS 4303) which have grounded inverting inputs, which act as current-to-voltage converters. The outputs of the two trans-impedance amplifiers are connected to the inputs of a differential amplifier (ADA4927). The output of the differential amplifier is the difference in the outputs of the two amplifiers, which is proportional to the instantaneous difference in the current across the upstream and downstream electrodes. Since the current is inversely proportional to the impedance of the electrodes at constant voltage, the output voltage is proportional to the negative of difference in impedance between the two electrodes in the linear approximation (provided, as is the case here, the change in impedance is small compared to the absolute impedance). As the cell passes between the upstream pair of electrodes, the impedance between the first pair of electrodes increases, resulting in an decrease in the current in the first pair and a negative voltage difference across the differential amplifier. Subsequently, when the cell passes through the downstream pair of electrodes, the impedance between the second pair of electrodes is larger, and there is a decrease in the current across the third pair of electrodes and a negative output voltage from the differential operational amplifier. This provides a wave from shown schematically in Fig. 5(b), and the amplitude of this voltage signal is proportional to the change in the impedance when the cell passes between the electrodes. Rather than report the actual impedance values, it is sufficient to correlate the output voltage of the differential amplifier with the size and properties of the particle passing through, since this remains the same for a given electrode and circuit configuration. Therefore, the impedance here is reported in mV, which is the output of the circuit shown in Fig. 5(a).

The lock-in amplifier for the two-dimensional impedance measurement was designed by Logic fruit and MicroX Labs on a Xilinx Vertex-7 FPGA. The lock-in amplifier has a two-output channel, and each channel can generate a combination of two frequencies ranging from 100 kHz to 4 MHz. More details regarding the lock-in amplifier are provided in appendix B.4. The sensitivity of the impedance sensor depends on the ratio of the volume of the cell and the volume of the detection chamber. Our sensor has a detection volume of 30*μ*m × 35*μ*m × 45 *μ*m, which is about 1.25 times smaller than the conventional Coulter impedance system. The sensitivity of the impedance chamber can be increased by reducing the measurement volume, but it is difficult to reduce the minimum dimension below about 30 *μ*m due to the danger of clogging of the microchannel. Another method is to increase the voltage in order to get a higher signal-to-noise ratio. In our case, an AC signal with 15 V peak-to-peak was used to increase the sensitivity of the signal. This enables us to detect particles as small as 2 *μ*m, as discussed in the validation in appendix C.

## Results

Before the cartridge was ready for the sample preparation the wash buffer was used to clean the interconnects, the mixing chambers and the reservoirs to avoid any clogging inside the impedance sensor. The composition of the wash buffer is 0.5% (w/v) sodium hypochlorite and 0.2% (w/v) of sodium hydroxide. Thereafter, the cartridge was dried into the vacuum desiccator for 24 hours before handling any blood sample. Six different blood samples were used from donors to validate the sample preparation inside the cartridge. The same cartridge was used to study the six different blood samples to avoid any variation which may arise due to any misalignment of layers during the fabrication process. Blood samples were obtained from healthy donors with informed consent after the approval of NAL health center research Ethics Committee. 3 ml of blood was collected into VACUTAINER (K2 EDTA 5.4 mg) tube. Following the blood collection, the EDTA tubes were kept on a roller for continuous mixing of the blood and the EDTA salt. All the sample preparation was done within four hours after taking the venous blood from the donor.

### RBC & Platelet enumeration

**Figure 6:**
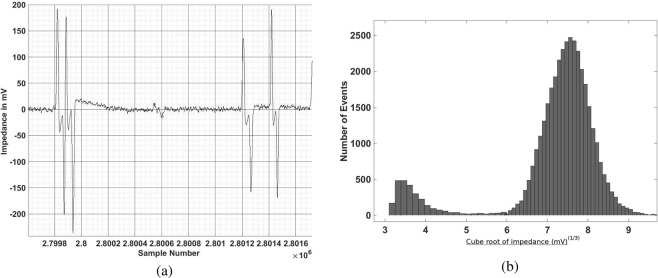
A sample of the output signal (**a**) and a histogram of the count as a function of the cube root of the impedance (**b**) for the enumeration of RBCs and platelets.

**Figure 7:**
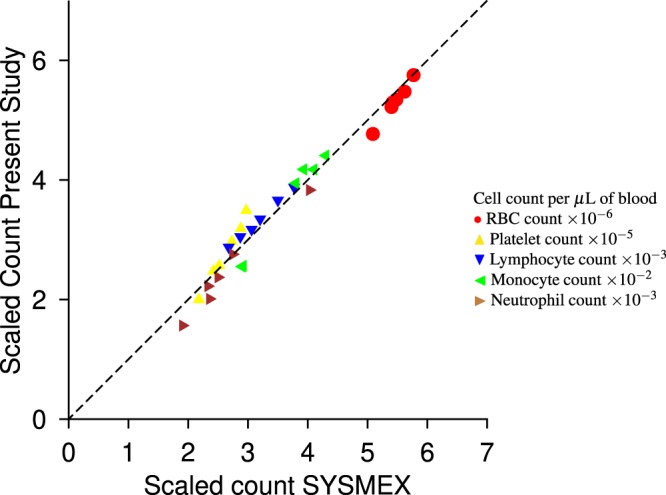
The scaled RBC, platelet, lymphocyte, monocyte and neutrophil counts determined from the commercial SYSMEX hematology analyser (*x* axis) and the present study (*y* axis). The two are equal along the dashed line.

After the sample processing discussed in section 2.2, the sample is passed through the sensor, with input voltage of 15 V peak-to-peak with frequency 2 MHz, and impedance measured at the same frequency. Figure 6(a) is a sample output signal, which clearly shows the passage of four RBCs and one platelet. Figure 6(b) is a histogram of the cell count vs. cube root of the impedance. This histogram clearly indicates two distinct populations of cells, and the distinction between the RBCs and the platelets is clearly visible, enabling the enumeration of these separately. The individual counts for the RBCs and platelets, scaled by 10^6^ and 10^5^ respectively, for the six samples are shown in Fig. 7. The *x* axis is the count obtained by the commercial SYSMEX hematology analyser, and the *y* axis is the result from the present study; the two counts are equal if they lie along the dashed line. Figure 7 shows that there is very good agreement between the results from the hematology analyser and the present study. The *R*^2^ measure (coefficient of determination) is 98.89% for the RBC enumeration and 99.02% for the platelet enumeration, indicating an excellent linear fit.

### WBC enumeration

**Figure 8:**
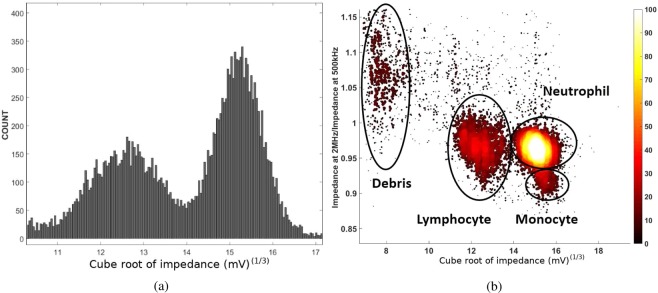
The histogram of the WBC count as a function of the cube root of the impedance at 500 Hz (**a**) and a scatter plot of the opacity as a function of the impedance.

The histogram for the WBC count as a function of the impedance at 500 kHz, shown in Fig. 8(a), reveals two distinct populations. Impedance measurements at two different frequencies, 500 kHz and 2 MHz, are carried out in order to distinguish between the three different WBC sub-populations, the lymphocytes, monocytes and neutrophils. The opacity is defined as the ratio of the two impedances. The plot of the opacity vs. cube root of impedance at 500 kHz clearly reveals three different populations, along with debris that has a very low impedance value due to RBC lysis, as shown in Fig. 8(b). Using opacity, it is possible to distinguish neutrophils and monocytes, which are indistinguishable in a single frequency impedance measurement.

The comparison between the present results and the commercial SYSMEX hematology analyser are shown in Fig. 7. Excellent agreement is observed even for the different WBC sub-populations, and the *R*^2^ measure is 99.54% for the neutrophil sub-population, 98.29% for the monocyte sub-population and 96.96% for the lymphocyte sub-population. Thus, we are able to achieve three-part WBC counting with determination coefficient of 97% or higher for all the sub-populations. The distinction of three sub-population of cells matches or exceeds that in literature^33^.

## Conclusion

This paper reported a fully automatic sample preparation inside the disposable cartridge where the three-part differential count along with the platelet count shows and excellent linearity with the FDA approved hematology analyzer. The cartridge, the size of a credit card, is fabricated using PSA tapes, PMMA sheets and PTFE/TPU membranes is easily fabricated using a commercial low-power laser cutter. The fabrication process is amenable for roll-to-roll manufacturing which is a low cost process; there are not complicated and expensive microfabrication procedures involved. A proprietary fabrication process has been developed for making the sensor using electrode deposition, electrode alignment and wafer bonding; since many of the processes are common to electronic chip manufacturing, this can easily be scaled up for low-cost commercial production. All of the pneumatic pumps, valves, controllers and electronics for sample preparation and signal processing are contained in a low-cost controller with a footprint of 15 cm × 11.5 cm and a height of 10 cm. The 7 layer PCB stack for signal processing was developed in-house, and is significantly smaller in size and lesser in cost than standard laboratory devices for impedance measurements. The results for enumeration of all of the cell types are in agreement with those of a standard commercial hematology analyser. Thus, this study has successfully demonstrated the feasibility of a portable low-power low-footprint chip-based device, incorporating a range of technologies, for autonomously carrying out a sophisticated multi-parameter diagnostic tests.

### Valves and pumps

**Figure 9:**
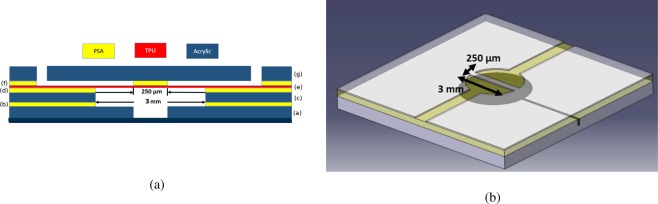
Cross section (**a**) and isometric view (**b**) of the valve structure depicting fluidic barrier of width 250 *μ*m with valve seat of diameter 3 mm (**b**). The labeling of the layers in sub-figure (**a**) corresponds to the labeling in the burst view in Fig. 2.

The design of the normally closed valve used in our disposable cartridge is shown in Fig. 9. The normally closed valve system^34–37^ for metering of the samples and the reagents are chosen in the disposable cartridge over the Quake valve^38^ because former requires less pressure to close the valve for a given pressure of the on-chip membrane pump. The thumb rule to operate the Quake valve is the pressure for closing the valve made of the soft membrane should be at least three times compared to the fluidic pressure in the channel to prevent any leakage, while in the normal close valve requires a fluidic pressure that is the same as the pressure applied on the soft membrane. Our disposable cartridge is operated with a differential pressure of ±50 kPA for opening and closing of the valve, and so a normally closed valve is preferred in this application.

In the valve layout in Fig. 9, the bottom PMMA layer of thickness 1 mm contains the air lines used for actuating the valves. Layer (c), made of 1 mm thick PMMA sheet, is used for creating extra depth and space for TPU membrane deflection, and the PSA layer (b) is used for bonding the two PMMA layers. Layer (d), made of PSA tape, is used as an adhesive layer for bonding TPU membrane with acrylic sheet. Layer (e) is TPU membrane, which is deformed when pressure is applied on it to open and close the valves needed for metering of samples and the reagents and for precise volume dispensing of the reagent stored in the reservoir. The valve seat has a diameter of 3 mm where pressure is applied. Sufficient depth and diameter of the access holes ensures proper bending of the TPU membrane when negative pressure is applied. Details of actuation pressure required for opening and closing of valves of different barrier length and width can be found in^39^. The TPU membrane (layer (e)) is initially bonded with the barrier in the PSA layer (f) that contains the fluidic channels with a barrier of width 250 *μ*m. Before the cartridge is ready for sample preparation, the cartridge was interfaced with the KNF pump and the negative pressure of −70 kPa was applied for more than 10 minutes on all the valves to break the adhesive bonding between TPU membrane and the fluidic barrier. After this pre-treatment, the membrane can be reversibly opened/closed by applying negative/positive pressure of 50 kPa.

**Figure 10:**
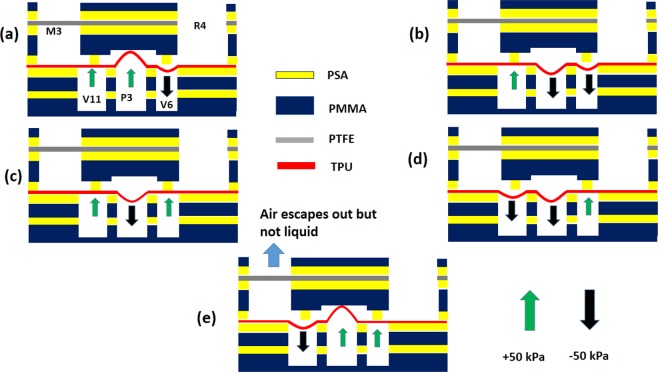
The principle for the on-chip membrane pump shown, as an example, for the pump P3 operating between valves V6 and V11 for pumping fluid from reservoir R4 to mixing chamber M3 in Fig. 3. The sequential opening and closing of valve is shown in the above figure (**a**) to figure (**e**). The PTFE membrane shown in gray vents the air, while preventing liquid escaping from the mixing chamber. The figure is not to scale in the horizontal direction, and the length of the channels between the valves, pump, mixing chamber and reservoir are significantly reduced in the representation.

The schematic of the on-chip membrane pump, shown in Fig. 10, is very similar to that for the valve. The principle of the membrane pump is shown sequentially in Fig. 10 for the pump P3 between the reservoir R4 and the mixing chamber M3 through valves V6 and V11 in Fig. 3. For ease of representation, the length of the channels between the valves, pump, reservoir and mixing chamber are compressed in the horizontal direction, and the figure is not to scale. First, a negative pressure is applied to TPU membrane below the upstream valve and the central chamber, to draw fluid into the central chamber. The upstream valve is closed, the downstream valve is opened and a positive pressure is applied to the central chamber to pump the liquid downstream. The time to complete the one stroke is 500 ms, and the amount of fluid transferred in one stroke is 9 *μ*L. The maximum equilibrium pressure measured from the fluid output port is 30 kPa.

**Figure 11:**
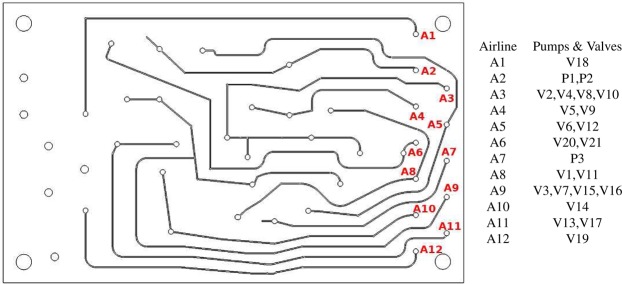
Schematic of the airlines in the bottom PMMA layer (**a**) in Fig. 2 which are used for controlling the valves and the pumps on the left, and the different pumps and valves controlled by the different airlines on the right.

For operating the 3 pumps and 20 valves, a total of 12 air lines of cross section 0.3 mm × 0.3 mm are engraved in layer (a) of Fig. 2, and access holes are made in layer (b) made of PSA tape. The layout of the airlines, and the valves and pumps controlled from the individual airlines, are shown in Fig. 11.

**Figure 12:**
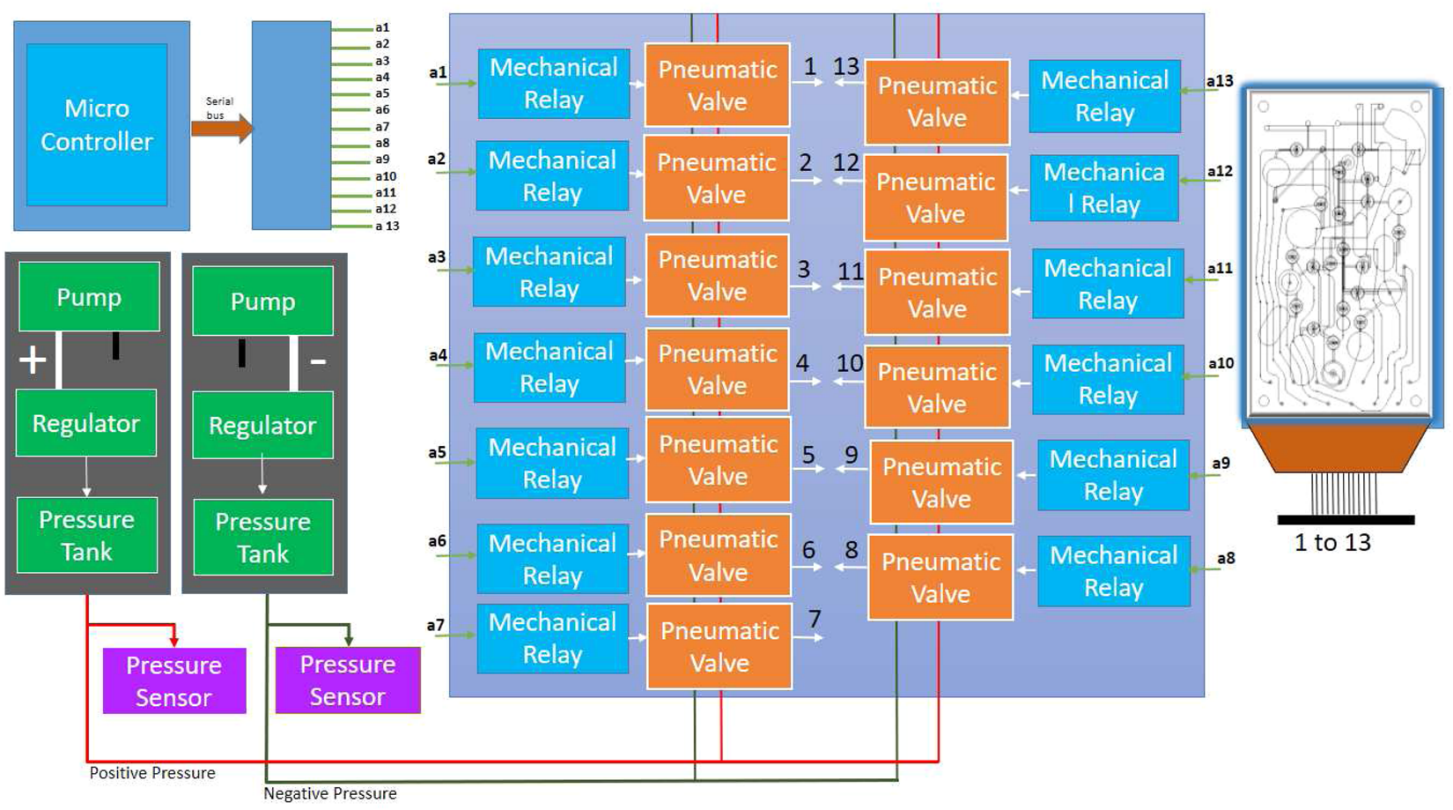
Block Diagram for controlling of the microfluidic valves and pumps. The pneumatic valve is connected with positive and negative pressure chamber and all 12 access holes are connected with the common connector of the 3-way solenoid valve from the bottom through 2 mm (ID) tube. The pressure in each 12 holes can be switched independently between + 50 kPa and −50 kPa.

The airlines A1-A12 are connected to three-way solenoid valves (XValve 912-000001-003) manufactured by Parker which are used for switching pressure between + 50 kPa and −50 kPa. All the solenoid valves are connected to mechanical relays manufactured by HUIGANG (HRS2H-S-DC5V) which are switched on and off using a micro-controller. Two KNF (NMP830KNDC) pumps generate positive and negative pressure. The positive and negative pressure is regulated using two pressure regulators from SMC Pneumatics. The block diagram for controlling the microfluidic pumps and valves is shown in Fig. 12.

### Sensor fabrication

Microfluidic devices were fabricated using photolithography techniques, and a cost effective approach was made throughout the fabrication process. A single-side polished SiO_2_ coated Si wafer (thickness ∼300 *μ*m) and borosilicate glass wafer (thickness ∼500 *μ*m) of diameter 50.8 mm were selected to fabricate the Microfluidic Impedance chip. A commercially available adhesive material (Perminex-2015, Microchem) negative tone photoresist and developer (PGMEA, Microchem) were used. There are three processes in the fabrication process, microelectrode fabrication, fluidic inlet/outlet ports, and micro fluidic channel fabrication followed by aligning and bonding of two wafers.

#### Sputtered patterned microelectrode fabrication using liftoff process

**Figure 13:**
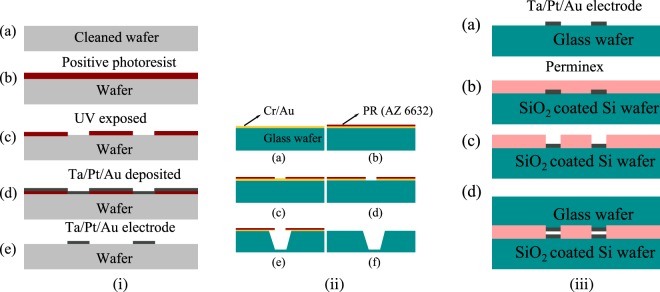
(**i**) Fabrication of microelectrodes by photolithography: (**a**) wafer clean through ultrasonication, (**b**) spin coating of positive photoresist on glass wafer, (**c**) UV light exposed by keeping electrode mask, (**d**) sputter deposition of Ta/Pt/Au on the top of the exposed and developed photoresist, (**e**) microelectrodes formed after liftoff process; (**ii**) Bonding: (**a**) microelectrodes formed on the top glass wafer, (**b**) perminex coated on the bottom wafer, (**c**) cross-linking of perminex to form microchannel, and (**d**) a sealed microfluidic channel formed by aligning and bonding of two wafers as shown in (**a**) and (**c**); (iii) Etching: (**a**) Cr/Au thin film sputter deposited on glass wafer, (**b**) spin coating of positive photoresist on Cr/Au coated glass wafer, (**c**) UV exposed with hole mask, (**d**) Cr/Au removal at drilling locations, (**e**) HF etching on patterned hole, and (**f**) 90% partial hole formed on glass wafer after complete removal of PR and Cr/Au film.

The first step is to fabricate patterned microelectrodes on both Si and glass wafers. The fabrication of these electrodes was carried out using the lift-off process as shown schematically in Fig. 13(i). A photolithography process (OAI, USA) was carried out to build a mold for the desired microelectrode fabrication. After completing the process, metal electrodes were deposited on a patterned mold wafer using UHV sputtering system with a base pressure around 1 × 10^−8^ torr. The deposited metal electrodes consist of three-layer stacks of Ta/Pt/Au with individual layer thickness around 20 nm/150 nm/100 nm, respectively. Here, Ta was used as an adhesive layer because the platinum did not adhere to glass and SiO_2_ substrates. After completing the deposition process, wafers were cleaned with appropriate cleaning agents to form 30 *μ*m wide and 30 *μ*m edge to edge spacing between two microelectrodes.

#### Inlet/outlet ports through wet etching process

There are several reported techniques to drill holes on glass wafers, such as mechanical drilling^40,41^ laser drilling^42,43^ and drilling through wet chemical etching^44–46^. Both high speed and laser drilling are prone to formation of micro cracks at the edges, which may lead to crack propagation through the channel line while bonding at higher pressure^47–50^. A femto-second laser could be used, but it is very expensive for batch fabrication. Stress-free drilling can be made through wet etching, as it dissolves the material at the area of contact between glass and the etchant solution. Here, a wet etching technique was used to drill a hole of diameter 1 mm on a borosilicate glass wafer, as shown in Fig. 13(ii).

Wafers were thoroughly cleaned with acetone and IPA by ultra-sonication and high purity nitrogen gas was used to dry the wafers. For dehydration baking, wafers were kept on the hot plate at 150 °C for 10 min and were allowed to cool down to room temperature. Cleaned glass wafer was coated with a thick Cr/Au (75 nm/800 nm) film using Ultra High Vacuum (UHV) sputtering system with the base pressure of 5 × 10^−8^ torr. After the deposition, a layer of positive photoresist (PR AZ6632- Microchem) was coated on the top of Cr/Au film. The wafer was photolithographically patterned to remove the positive photoresist at the drilling locations on the wafer and then baked on the hot plate at 125 °C for 35 mins. After curing, the whole wafer was attached to 2″ diameter wet processing wafer chuck (AMMT Germany) made of PEEK (Polyether ether ketone) to carry out the etching process. Initially, Cr/Au was removed at the drilling locations using respective agents such as Cr etchant and aqua regia (*H*_2_*O*: *HCl*: *HNO*_3_–10:9:1). Then, the whole setup was immersed into a Teflon beaker containing 48% HF solution for 35 mins to make a through hole with a depth of around 90% of the total thickness of the wafer. The schematic of the etching mechanism is shown in Fig. 13(ii). Finally, through holes were made using diamond scribe pencil (SPI 06002-AB).

#### Microfluidic channel fabrication

The fabrication steps for the microchannel fabrication within the impedance sensor are shown schematically in Fig. 13(iii). The creation of the fluidic channel involves optimization of spin coating parameters to obtain desired thickness and uniform perminex film, tuning the photolithography parameters to obtain the well defined fluidic channel and a custom made setup for bonding. The microfluidic channel was formed using a photoimageable adhesive material which was spun on the top of microelectrode patterned Si wafer using a spin coater. The spin coater parameters were varied as provided in Table 1, to obtain the information on the thickness of the perminex layer. From Table 1, it is clear that the thickness of the perminex can be varied from 18 to 48 *μ*m by tuning the second ramp rate. To evaporate the solvent, the perminex coated wafer was then kept on the hot plate for pre-baking at 95 °C for a duration of 5–7 minutes. The pre-baked wafer was then exposed to UV light under the mask to obtain a defined channel pattern. A bake step after exposure was then carried out on a hot plate at 70 °C.

**Table 1:** Parameters for perminex spin coating.

	Factor	18 *μ*m	24 *μ*m	30 *μ*m	48 *μ*m
First ramp	Speed	1000	1000	1000	1000
	Acceleration	200	200	200	200
	Time	10 s	10 s	10 s	10 s
Second ramp	Speed	3000	2000	1500	1000
	Acceleration	1000	1000	800	800
	Time	30 s	30 s	30 s	30 s

The details of the optimisation of the etching process, low temperature adhesive wafer bonding process, evaluation of the bonding process and the testing of fluid flow through the sensor are provided in the Supplementary Information section.

#### Impedance measurement

A custom lock-in amplifier was designed on a Xilinx Vertex-7 FPGA. The board has two output channels. Each channel can generate a combination of two frequencies needed for cell counting. The lock-in amplifier was interfaced with the analog domain by using high-speed data converters (DAC3282, TI) operating at 625 Msps. The sampling of data from the impedance sensor after signal conditioning was done at 40 Msps. The signal was modulated at 2 MHz for the impedance measurement of the beads. In our case, 15 *V*_*pp*_ signal was applied to the signal electrodes for the impedance measurement. The average velocity of the fluid inside the impedance sensor is 0.37 m/s for a flow rate of 30 *μ*L/min. The choice of the low pass filter cut off frequency for demodulation of the signal depends on the maximum possible velocity of the cell inside the sensor and the electrode dimension (in the direction of flow). The maximum possible velocity was assumed to be double of the average velocity inside the microfluidics, assuming laminar flow in the sensing region.

A simple MATLAB script was used to find the minimum low pass filter cut off frequency needed for a given flow rate and electrode geometry. This was done by fitting the Fourier series on the impedance signal and thereafter the fitted signal was passed through a 20 tap FIR low pass filter to find the minimum cut off frequency needed to preserve the signal amplitude and shape for the given flow rate of 30 *μ*l/min and the electrode geometry. The under-sampling of the data after demodulation was done at 250 ksps for the impedance-data recording. All the files are recorded in the binary format to minimize the memory space required for data storage. The low pass filter cut-off frequency employed in our case is 50 kHz, which is five times more than the calculated minimum cut off frequency required for the demodulation of the signal.

**Figure 14:**
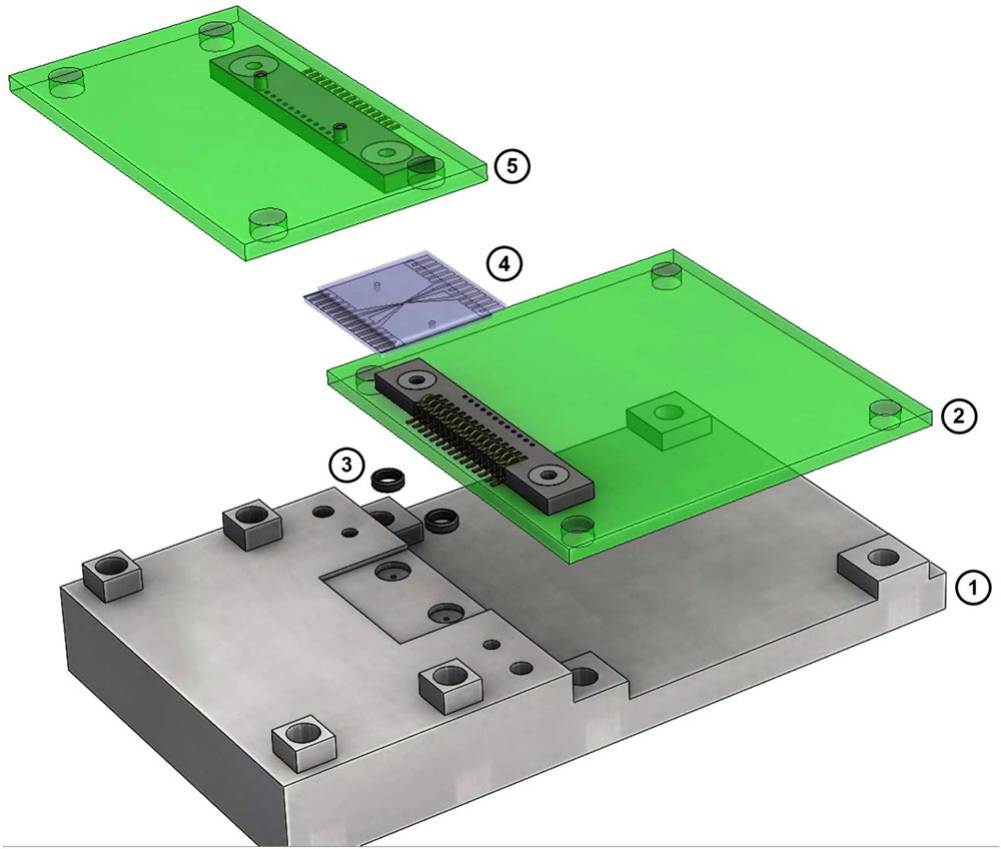
Overview of electrical connections to the microfluidic impedance sensor: (**1**) 3D printed holder (**2**) PCB with Samtec connector for top electrode (**3**) O-rings, (**4**) microfluidic impedance sensor, and (**5**) PCB with Samtec connector for bottom electrode.

The front-end electronics and the impedance sensor are all mounted on an acrylic holder (machined using 5 axis Datron, C5) to make the electrical connection short to minimize the parasitic capacitance and noise coupling between the circuits as shown in Fig. 14. The two PCBs, which are mounted on the PMMA board, have Samtec spring connectors(SEI-115-02-GF-S-M-AB-TR), which makes electrical contact with the impedance sensor. The fluidic inlet and outlet holes of diameter 1.6 mm were drilled into the holder. The plastic tubing having an inner diameter of 0.5 mm with an outer diameter of 1.5 mm was glued into the holes for the fluidic connection. Two O-rings, each having an inner diameter of 1 mm and an outer diameter of 3 mm, were positioned into the drilled slot of the fluidic path with depth 0.3 mm and thereafter the impedance sensor was tightly clamped on the holder using screws and metal bar made of brass. All the events from the captured data were recovered by finding the maxima and minima of an impedance signal.

### Sensor validation

**Figure 15:**
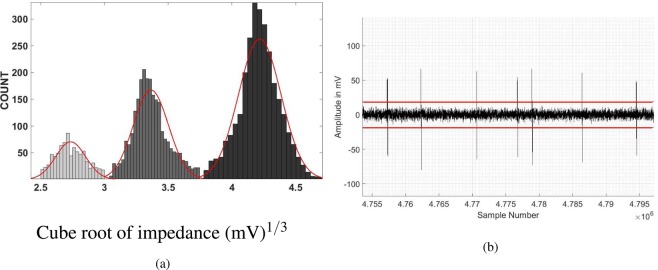
The histogram of the cube-root of the impedance of the 3,4 and 5 *μ*m polystyrene particles passing through the impedance sensor (**a**); and the voltage signal and noise from the passage of particles of diameter 3 *μ*m in the impedance sensor (**b**). The measurement is carried out at a frequency of 2 MHz, and the red lines in sub-figure (**b**) show the expected signal level for particles of diameter 2 *μ*m if the diameter is proportional to the cube root of the impedance.

A mixture of polystyrene particles of size 3 *μ*m, 4 *μ*m, and 5 *μ*m (Sigma Aldrich) were suspended into the PBS(1×) solution containing 0.1% (v/v) Tween 20 and 7% sucrose solution which has the same density as the particles. The measured conductivity of the solution was 1.0 S/m. The bead solution was loaded into 1 ml BD syringe and pushed through an impedance sensor at a flow rate of 30 *μ*L/min. The cells were passed through the sensor, and the histogram of the cube-root of the impedance at frequency of 2 MHz is shown in Fig. 15(a). The Gaussian fits for the cube-root of the impedance are also shown. Three clear populations are visible in the histograms, indicating that it is possible to clearly distinguish between particles of size 3,4 and 5 microns. The values the cube root of the impedance at the maxima of the Gaussian fits are in the ratio 3:4:5, confirming that the cube root of the impedance is proportional to the particle size, or the impedance is proportional to the particle volume. Thus, the particle size can be determined based on the calibration of the impedance. In fact, the impedance sensor accurately predicts not just the average particle size, but the standard deviation of the size distribution as well. The standard deviation and the Coefficient of Variation (CV) of the distributions determined using our sensor are compared with the manufacturer specifications in Table 2. There is quantitative agreement between the results from the impedance sensor and the manufacturer specifications.

**Table 2:** The standard deviation and the Coefficient of Variation (CV) from the manufacturer specification compared with the experimental result for the validation tests on the impedance sensor.

Diameter	Manufacture’s data			Experimental result		
	Mean	Std. dev	CV (%)	Mean	Std. dev	CV %
*μ*m	3	0.12	4	3	0.132	4.4
*μ*m	4	0.08	2	4	0.132	3.3
*μ*m	5	0.1	2	5	0.16	3.2

The above analysis demonstrates that it is possible to unambiguously detect particles as small as 3 *μ*m, that is, the size of platelets, and that it is possible to distinguish particles that differ in diameter by 1 *μ*m. In order to determine the detection limit, the particle counting was performed using a suspension of only 3 *μ*m particles suspended into the PBS(1×). A sample of the voltage signal is shown in Fig. 15(b). The >50 mV peaks in the voltage signal indicate the passage of 3 *μ*m particles greater, whereas the noise level is less than 10 mV. The average voltage signal for the 3 *μ*m particles is about 62.5 mV. If we consider the particle size proportional to the cube root of the impedance, the voltage level expected for 2 *μ*m particles is about 18.5 mV. This voltage level is clearly significantly larger than the noise level in Fig. 15(b), indicating that the lower limit on the particle size that can be counted by the impedance sensor is about 2 *μ*m.

## Supporting Information

Supporting Information
