# Author Correction: A cartridge based Point-of-Care device for complete blood count

**DOI:** 10.1038/s41598-020-76738-3

**Published:** 2020-11-16

**Authors:** Usama Abbasi, Prasanta Chowdhury, Sasikala Subramaniam, Prakhar Jain, Nitin Muthe, Faisal Sheikh, Subham Banerjee, V. Kumaran

**Affiliations:** 1 MicroX Labs, Society of Innovation and Development, Indian Institute of Science, Bangalore, 560012 India; 2 Department of Chemical Engineering, Indian Institute of Science, Bangalore, 560 012 India; 3 CSIR-National Aerospace Laboratory, HAL Airport Road, Bangalore, 560017 India

Correction to: *Scientific Reports* 10.1038/s41598-019-54006-3, published online 09 December 2019

This Article contains typographical errors.

In the Introduction section,

“Across a microchannel width of W ∼ 1 m, the diffusion time is (W^2^/D) ∼ 10^3^–10^6^ s.”

should read:

“Across a microchannel width of W ∼ 1 mm, the diffusion time is (W^2^/D) ∼ 10^3^–10^6^ s.”

In the Cartridge section, the three references to “appendix A” should read “the section on Valves and pumps.”

Under the subheading ‘Metering of blood sample’, the reference to “sections 2.2 and 2.3” should read “the section on Sensor Fabrication”.

In the Sensor section, the reference to “appendix B” and to “appendix B.4” should read “the section on Sensor Fabrication.”

Additionally, in this section, the reference to “appendix C” should read “the section on Sensor validation.”

In the Results, under the subheading ‘RBC & Platelet enumeration’, the reference to “section 2.2” should read “the section on Sample preparation for RBC & platelet enumeration.”

Finally, in the legend of Figure 8,

“The histogram of the WBC count as a function of the cube root of the impedance at 500 Hz (**a**) and a scatter plot of the opacity as a function of the impedance.”

should read:

“The histogram of the WBC count as a function of the cube root of the impedance at 500 kHz (**a**) and a scatter plot of the opacity as a function of the impedance (**b**).”

