# Supplementary material for: A cartridge based Point-of-Care device for complete blood count
Source: Sci Rep. 2019 Dec 9;9:18583. doi: 10.1038/s41598-019-54006-3 (PMC6901560; doi:10.1038/s41598-019-54006-3)
Supplement: Supplementary file 1 — Supporting Information [file 41598_2019_54006_MOESM1_ESM.pdf]

Supplementary Information
A cartridge based Point-of-Care device for complete blood count

Usama Abbasi[1], Prasanta Chowdhury[3], Sasikala Subramaniam[1], Prakhar Jain[1], Nitin Muthe[1], Faisal Sheikh[1], Subham Banerjee[1] V. Kumaran[2,*],
[1]MicroX Labs, Society of Innovation and Development, Indian Institute of Science, Bangalore 560012, India,
[2]Department of Chemical Engineering, Indian Institute of Science, Bangalore 560 012, India,
[3]CSIR-National Aerospace Laboratory, HAL Airport Road, Bangalore 560017, India,
*kumaran@iisc.ac.in

Supplementary methods

Optimisation of etching process

In general, the hydrophilic nature of Cr/Au film enables HF molecules to diffuse into the glass and cause the development of pits. There are several reports of the creation of pits during the wet etching process with Cr/Au layer thicknesses. Among them, defects and micro-creep also may cause the generation of pin holes which are formed during the sputter deposition of Cr/Au layer. Figure 1 (a) and (b) show the pit formation on the glass wafer during etching in HF (48%) solution for a duration of 35 min. Here, the glass surface was masked with sputtered layers of Cr (75nm)/Au(400 nm) in combination with a photoresist (AZ 6632) layer of a thickness of 3.2 μm. Baking of photoresist layer may further reduce the diffusivity of HF molecules

due to the penetration and filling the cracks and also due to the change in surface phobicity from hydrophilic to hydrophobic in nature. With increasing the Au layer thickness from 400 nm to 800 nm and baking the PR layer at 125 ^{o}C for a duration of 25 min, no pits were found after the etching process as shown in figure 1 (c) and (d). The etching process was carried out for an optimized duration to obtain a depth of 450 μm with an etching rate of 12.8 μm/min. The profile of the etched hole is shown in figure 1 (e).

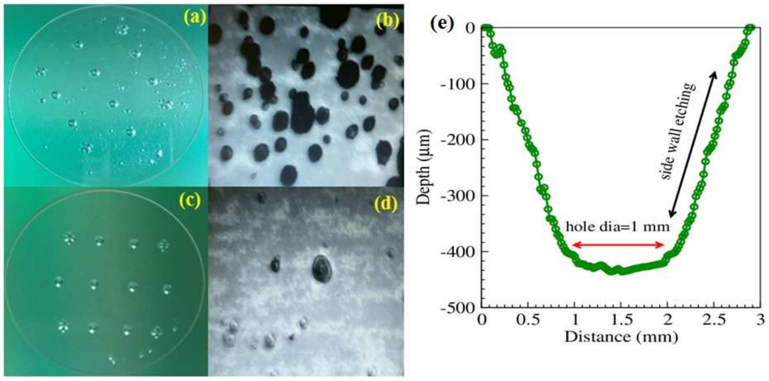

Supplementary Figure 1: Etching for inlet/outlet holes: (a) HF etching with 400 nm Cr/Au mask, (b) an optical image of the resulting glass wafer showing the formation of pin holes and pits, (c) HF etching with 800 nm thick Cr/Au mask, (d) an optical image of resulting glass wafer showing the pit-free surface, and (e) a depth profile measurement confirming that 90% partial hole formed after HF etching.

Low temperature adhesive wafer bonding process

The alignment and bonding of two wafers was carried out with an accuracy of $\pm$ 1 μ m using mask aligner(OAI, USA). The glass wafer, containing patterned microelectrodes and through holes, was softly glued on a 4 inch $\times$ 4 inch transparent glass plate and fixed on the mask holder slot. The SiO_2 wafer, containing both patterned electrode and perminex fluidic channel, was fixed on the wafer vacuum chuck holder. Both the wafers were manually aligned with an accuracy $\pm$1 μm and softly bonded through an adhesive layer. A bonded wafer was then transferred to a pressurized die to hold the wafer under a pressure of $\sim$ 10 to 50 N. The entire whole chuck was transferred

to a oven for soaking around at a temperature of 120 ^{o}C for an hour and cooled down to room temperature. A bonded wafer was then removed from pressurized holder and diced it to have individual chips of dimension 13 mm $\times$ 13 mm. Contact pads of both top and bottom electrodes were partially opened at the opposite edges of the chip for further electrical connections.

Evaluation of bonding quality

The bonding quality was evaluated based on its unbonded area on the wafer corresponding to applied pressure and temperature, though there is no literature available to the best of our knowledge. Initially, wafer bonding was carried out using a commercial aligner and bonder (AWB 04, AML, UK), where the temperature and pressure were applied as per the data sheet provided by the manufacturer. Initially, the temperature was ramped to the set value, i.e., 150 ^{o}C and the pressure was applied up to 4 kN for an hour, but this resulted in delamination. The reason could be the hardening of adhesive during the temperature ramping before the pressure is applied. In subsequent trials, where both temperature and pressure were applied simultaneously, misalignment was observed between the wafers. Then several attempts have been made to bond the wafer using our custom made pressurized die with varying pressure from 4 kN to 2kN and soaked at 110 ^{o}C in an oven under the pressurized condition and all attempts were resulted in squeezing of the channels. As given in table 1, the best results were achieved at low pressure (<100 N) and when soaked at a temperature of 120 ^{o}C for an hour. The snapshot of the bonded wafer is shown in figure 4 (a) of the paper. Figure 4 (b) of the paper shows the optical images of the channel along with aligned electrodes taken after sealing two wafers. The wafer was then further diced and the final chip of dimension 13 $\times$ 13 mm^2 was realized as shown in figure 4 (c) of the paper. Implementation of low-pressure bonding was applied for ten sets of the wafers and 50 devices were obtained for further testing in impedance cytometry.

Interconnection and flow test

To conduct the flow test, the micro impedance chip was mounted between two acrylic plates as shown in figure 2 (a). Inlet and outlet ports were sealed with O-rings to prevent any leakage at the interface between the chip and the top acrylic plate. The top plate was laser cut in such a way that inlet

Pressure	Temperature	Time	Bonder	Results
4KN	150	1 hour	AML	Delaminated
3KN	150	1 hour	AML	Misalignment
4KN	110	30 mins	in house	All squeezed
3KN	110	30 mins	in house	2 squeezed
<100 N	120	1 hour	in house	Good bonding

Supplementary Table 1: Results of different pressures and temperatures used in the bonding process.

and outlet ports of the sensors and the acrylic plate were exactly aligned. A "T" connector was used to connect the pressure sensor (Model No. ABP series (0 to 10 bar), Honeywell) and the MIC chip with the syringe pump for the pressure drop measurement between the inlet and outlet port of the MIC chip as shown in figure 2 (b). Filtered DI water was pushed through the syringe pump and the flow rate was varied from 10 μl/minute to 100 μl/minute.

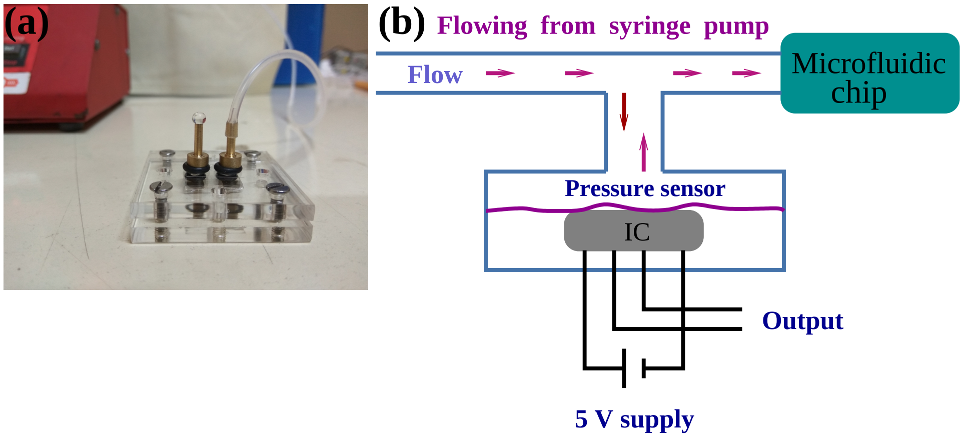

Supplementary Figure 2: Image (a) and schematic (b) of the setup for pressure and flow rate measurements.

The relationship between the pressure and flow rate is linear, as shown in figure 3. For a flow rate of 100 μl/min normally required for the PoC (Point of Care) devices, the pressure drop required is about 40 kPa, which can easily be attained using the micro-pumps required here. There was no

leakage from the device at a pressure as high as 100 kPa, which was attained by blocking the fluidic line.

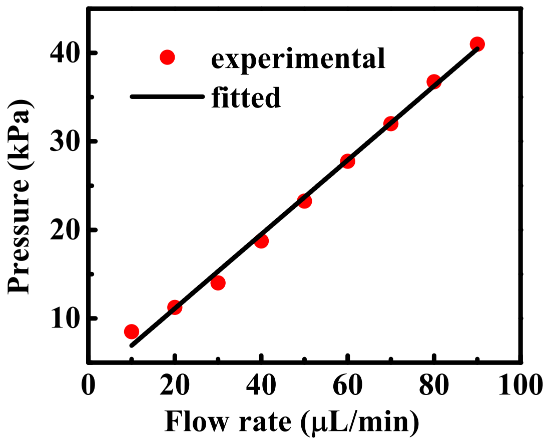

Supplementary Figure 3: Relationship between pressure and flow rate for the flow through the sensor.
